# Mapping the Protein Fold Universe Using the CamTube Force Field in Molecular Dynamics Simulations

**DOI:** 10.1371/journal.pcbi.1004435

**Published:** 2015-10-27

**Authors:** Predrag Kukic, Arvind Kannan, Maurits J. J. Dijkstra, Sanne Abeln, Carlo Camilloni, Michele Vendruscolo

**Affiliations:** 1 Department of Chemistry, University of Cambridge, Cambridge, United Kingdom; 2 Departments of Bioengineering, Stanford University, Stanford, California, United States of America; 3 Department of Computer Science, Vrije Universiteit, Amsterdam, The Netherlands; George Mason University, UNITED STATES

## Abstract

It has been recently shown that the coarse-graining of the structures of polypeptide chains as self-avoiding tubes can provide an effective representation of the conformational space of proteins. In order to fully exploit the opportunities offered by such a ‘tube model’ approach, we present here a strategy to combine it with molecular dynamics simulations. This strategy is based on the incorporation of the ‘CamTube’ force field into the Gromacs molecular dynamics package. By considering the case of a 60-residue polyvaline chain, we show that CamTube molecular dynamics simulations can comprehensively explore the conformational space of proteins. We obtain this result by a 20 μs metadynamics simulation of the polyvaline chain that recapitulates the currently known protein fold universe. We further show that, if residue-specific interaction potentials are added to the CamTube force field, it is possible to fold a protein into a topology close to that of its native state. These results illustrate how the CamTube force field can be used to explore efficiently the universe of protein folds with good accuracy and very limited computational cost.

## Author Summary

Modelling protein behaviour using computer simulations has progressively emerged in the last 50 years as a powerful strategy in structural and molecular biology. Over this period there has been a continuing interest in pushing the boundaries of this approach in terms of the size of the systems and the timescale of the processes that can be studied. Coarse-grained models offer in principle great opportunities in this context, but it has been extremely challenging to obtain force fields of accuracy comparable to that typical of fully atomistic models. We show here that the representation of protein molecules as self-avoiding tubes within the CamTube model enables the comprehensive, accurate and very fast exploration of the conformational space of proteins in molecular dynamics simulations. We illustrate in particular how the comprehensive mapping of the protein fold universe obtained using the CamTube model offers the possibility of analysing the behaviour of proteins in a wide range of non-native states.

## Introduction

The conformational space of proteins is made up by a vast number of structures [1,2,3,4,5,6,7], and yet, only a limited menu of protein folds appears to exist [8,9,10]. A better understanding of the structural properties of proteins underlying this remarkable organisation of their conformational space will undoubtedly facilitate computational studies of these molecules by helping reduce the number of degrees of freedom that are required in the simulations.

In this context, coarse-grained models of protein structure and dynamics provide several opportunities since their simple form makes it possible to reproduce generic features common to all proteins. Such coarse-grained approaches usually reduce the structural resolution by using a small number of interaction sites to represent a residue and by developing parameters based on various physical considerations [11,12,13,14,15,16,17,18]. A recently emerged class of coarse-grained approaches uses the so-called ‘tube’ model of a protein structure, whereby the polypeptide chain is represented as a thick tube that satisfies physically motivated constraints on its shape and curvature [19,20,21,22]. It has been shown that a tube-like description of proteins that includes energetic contributions from hydrogen bonding and hydrophobicity results in a free energy landscape that exhibits a variety of structures corresponding to the tertiary folds observed in the Protein Data Bank (PDB) [22]. It has also been found that when amino acid-specific interaction energies are incorporated in a tube model in place of generic hydrophobic terms it is possible to design sequences that fold into particular target structures [23,24].

Given the efficiency with which tube models can explore the conformational space of proteins and populate protein-like structures, it would be desirable to adapt these models to molecular dynamics simulations, thus extending their scope and public availability. In this paper we address this problem by presenting a version of the tube model, called CamTube, in which the tube requirements are implemented as a force field in the Gromacs molecular dynamics simulations package [18]. To illustrate the behaviour of the CamTube approach, we first reproduce the phase behaviour of a previously reported tube model using molecular dynamics simulations [22]. Then, we demonstrate that the CamTube model can reproduce the rich conformational space of a 60-residue polyvaline chain previously observed in all-atom simulations of this system [25]. Finally, we test current residue-specific properties of the CamTube model by folding GB3, a 56-residue mixed α-β protein.

## Results/Discussion

### Phase behavior of the CamTube polypeptide chains

In order to better understand how the energy terms in the CamTube force field affect the conformational space explored by a polypeptide chain, we carried out a series of simulations of a 60-residue polyvaline polypeptide chain (Val60) in which we varied the hydrophobic energy (*ε*_*W*_) and the curvature penalty (*κ*_*c*_) parameters while holding fixed the hydrogen bond energy and the temperature. Val60 serves as a suitable test case for the CamTube force field, since its conformational space was recently characterized by all-atom molecular dynamics simulations and found to cover a large variety of folds of small proteins [25].

**Fig 1:**
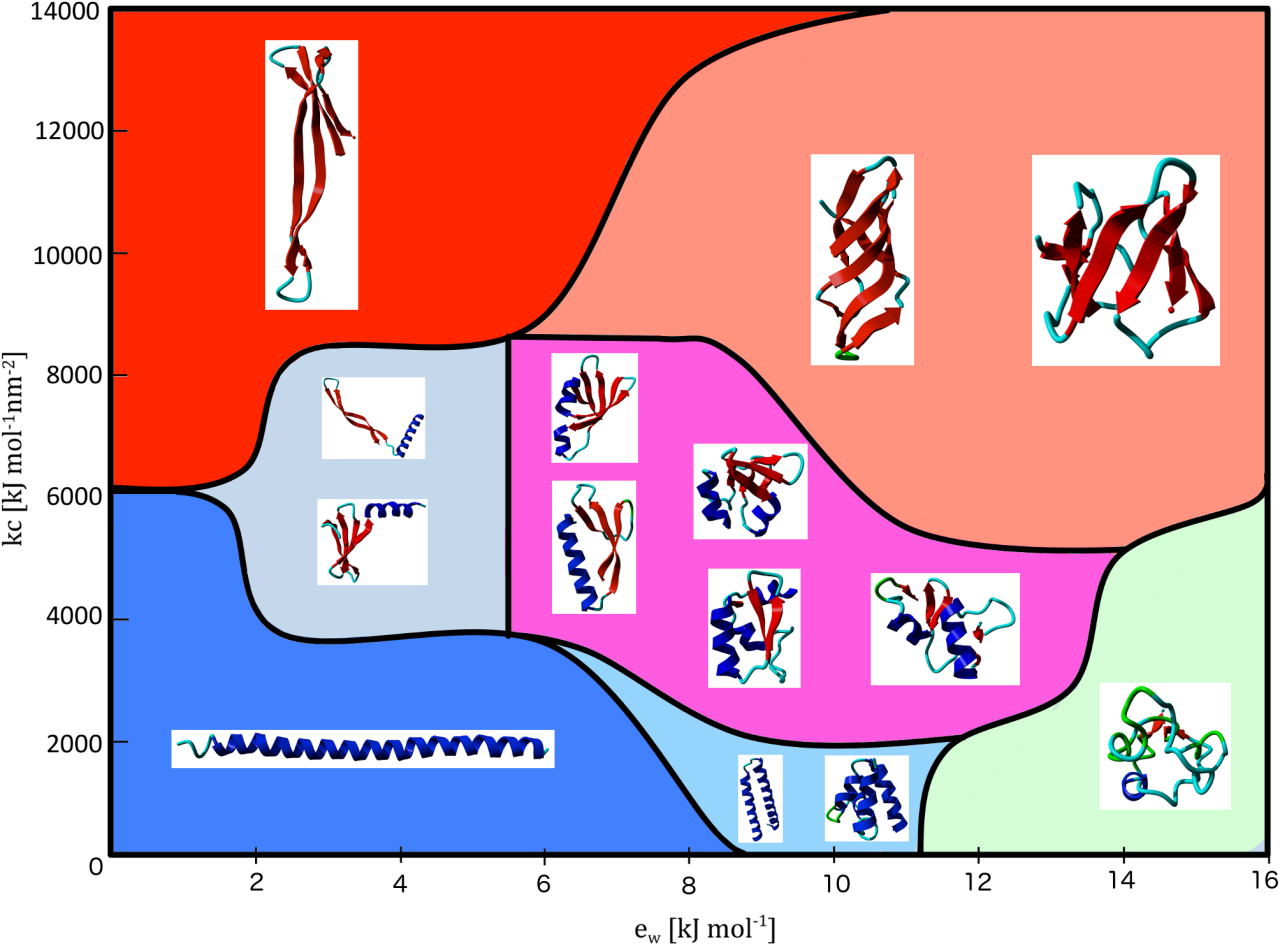
Phase diagram of Val60 in the CamTube force field. The phase diagram is shown as a function of the hydrophobic energy, *ε*_*W*_, and the curvature, *κ*_*c*_, parameters. Representative structures in each region of the parameter space are shown as insets.

At each *(ε*_*W*_, *κ*_*c*_ *)* point on a grid in the parameter space, a 100 ns simulation, which is long enough to allow the polypeptide chain to adopt a stable fold, was initiated from a random coil configuration of Val60. Upon varying *ε*_*W*_ and *κ*_*c*_, we observed a number of different states in which the structures adopted by the polypeptide chain change dramatically across boundary lines in the *(ε*_*W*_, *κ*_*c*_ *)* plane. The locations of these transitions define curves in the parameter space that yielded a phase diagram shown in Fig 1. When *ε*_*W*_ is sufficiently small, one obtains a phase with very few contacts between the Cβ atoms. These unfolded stable structures feature a long α-helix, a stranded β-sheet or coexistence of α-helical and β-sheet structural motifs without any tertiary contacts. When *ε*_*W*_ is sufficiently large, one finds a very compact stable globular state with featureless ground states and an extremely high number of contacts.

Between these two phases, a marginally compact phase emerges with complex tertiary folds found in natural proteins. Depending on the choice of the *kc* parameter this marginally compact phase samples a range of tertiary folds. When *kc* is sufficiently low, the sampled structures include a bundle of two, three or four α-helices. For intermediate range of *kc* values (2000 < *kc* < 8000), we observe a range of distinct supersecondary arrangement that contain both α-helical and β-sheet structural motifs. Lastly, for sufficiently large *kc* values, the CamTube force field samples β-barrel conformations with different number of β-strands.

The type of phase behaviour observed in Fig 1 is consistent with the results obtained originally for the tube model using a hydrophobic-polar (HP) interaction energy with explicit geometric terms in the energy function [22]. Thus, the CamTube force field is able to ‘presculpt’ the protein conformational space characteristic of the tube model in a molecular dynamics context by means of suitably tuned molecular dynamics potentials that mimic the effects of geometric constraints. This result provides further support for the suggestion that the range of folds accessible to proteins can arise from only few sequence-independent energy constraints imposed by the inherent geometric properties of polypeptide chains [20,21,22]. One should note, however, that even slight changes in these geometric features, as manifested by variations in *ε*_*W*_ and *κ*_*c*_ in Fig 1, can dramatically alter the free energy landscape and eliminate the rich repertoire of tertiary motifs that characterizes the library of natural protein folds.

### Conformational space of a CamTube polypeptide chain

In addition to validating the geometric parameters used in the CamTube model, we also probed in detail the conformational space described by the CamTube model. To understand how many independent structures can actually be explored with the CamTube force field, we carried out a molecular dynamics simulation of Val60 using *ε*_*W*_ and *κ*_*c*_ parameters that correspond to the centre of the α/β marginally compact phase (*ε*_*W*_ = 10.0 kJ mol^−1^ and *κ*_*c*_ = 2000 kJ mol^−1^ nm^−2^). As a sampling method, we used bias exchange metadynamics protocol [26] because of its ability to explore low probability regions of the conformational space and freely diffuse along CVs. Four CVs were chosen here, namely backbone dihedral angles, number of α-helical and β-sheet 6-residue fragments and the radius of gyration (see Methods).

**Fig 2:**
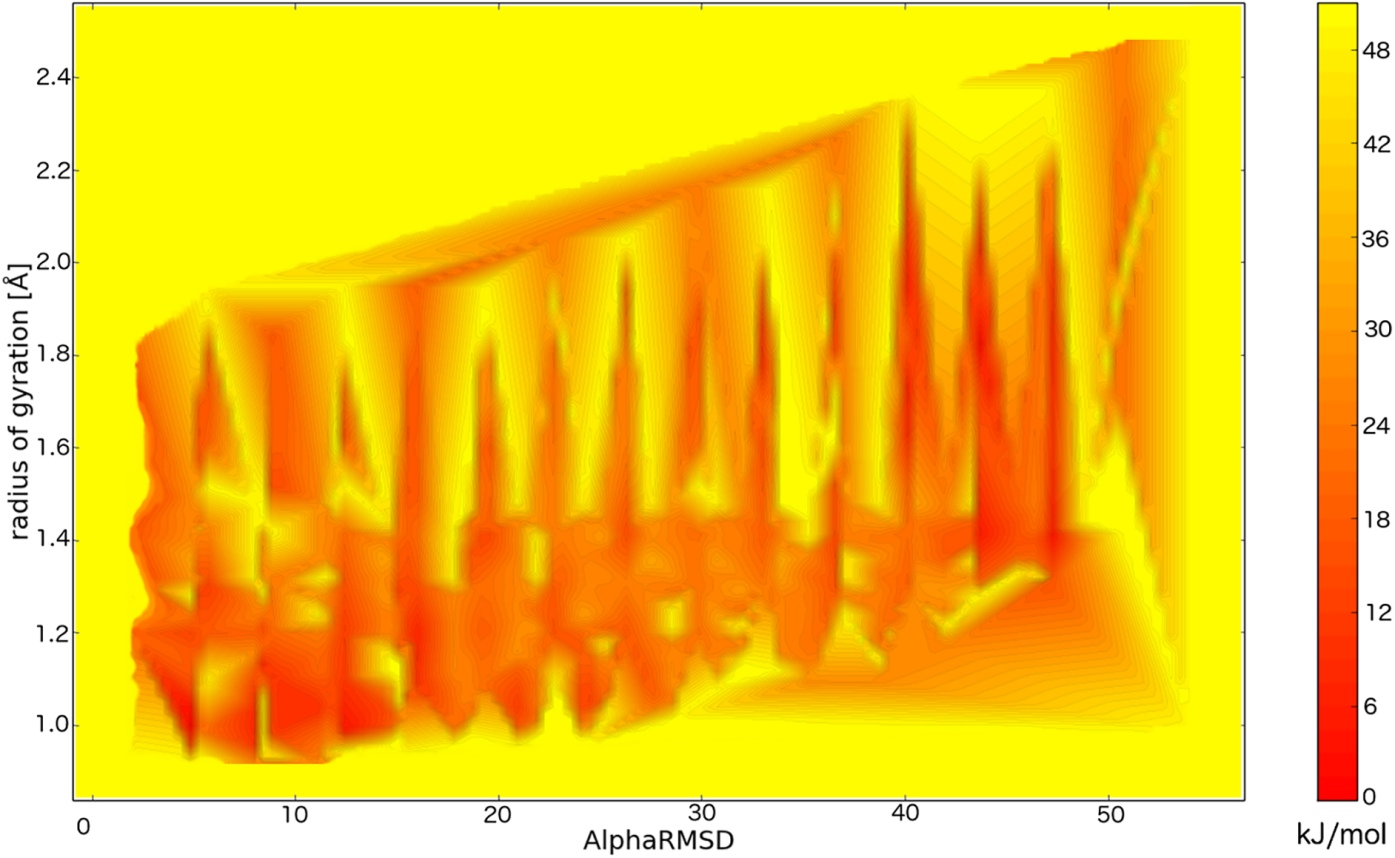
Free energy surface of Val60 in the CamTube force field. The x and y axes represent two CV variables: the number of α-helical six-residue-long fragments and the radius of gyration.

**Fig 3:**
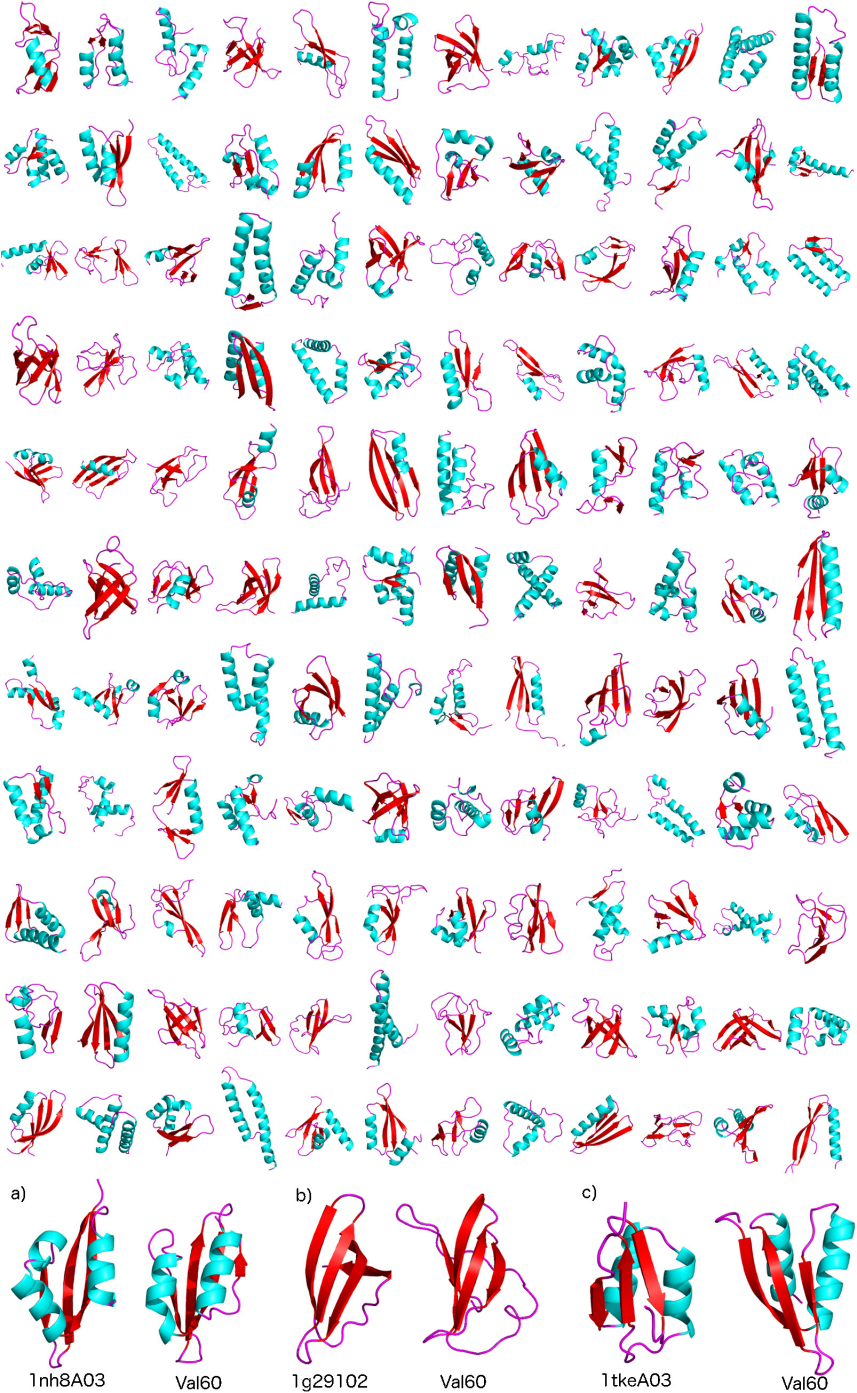
A repertoire of representative Val60 structures generated using the CamTube force field. A selection of 135 structures whose TM-score from respective CATH structures is larger than 0.4; a-c) examples of three CATH structures with their equivalent Val60 structures. CATH codes are given bellow the respective figures.

The reconstructed free energy landscape of Val60 as a function of the number of α-helical fragments and the radius of gyration is shown in Fig 2. We found that the free energy surface of Val60 features many conformations with similar free energies. Following a previously described procedure [27], the conformational space of Val60 was clustered in microstates (see Methods), which were then further analysed. To check how many known folds can be reproduced with the identified microstates, we used the CATH database [28]. This database is widely used in structural studies to classify protein folds and for the purpose of this study we selected all 265 CATH folds of the proteins containing between 40 and 75 amino acids (see Ref. [25] for the list of CATH structures). For each fold in the CATH subset, we searched in the set of the microstate conformations for its most similar structure as quantified by the TM-score [29]. We use the TM-score here because it is a measure sensitive to the global topology rather than to the local structural errors. This score lies in the [0,1] interval, with values above 0.4 indicating conformations with similar topologies, and values below 0.17 indicating conformations with different topologies [29]. For 135 out of 265 CATH structures, we were able to find at least one microstate with similar tertiary structure (TM-score > 0.4). All 135 Val60 structures are shown in Fig 3 together with three representative CATH structures. These results indicate that the conformational landscape described by the CamTube model exhibits free energy minima that can be explored efficiently by molecular dynamics simulations. Within these minima there are variety of tertiary structures that accurately reflect the diversity of protein folds observed in the PDB. Indeed, the microstates sampled from the metadynamics simulation of Val60 cover about half of all known classes of natural folds of proteins of similar size. It is important to emphasize that these folds have been selected here on the basis of the geometry requirements of the CamTube model and not on the physico-chemical properties of the amino acid sequence.

### Conformational space of a small globular protein populated using the CamTube force field

**Fig 4:**
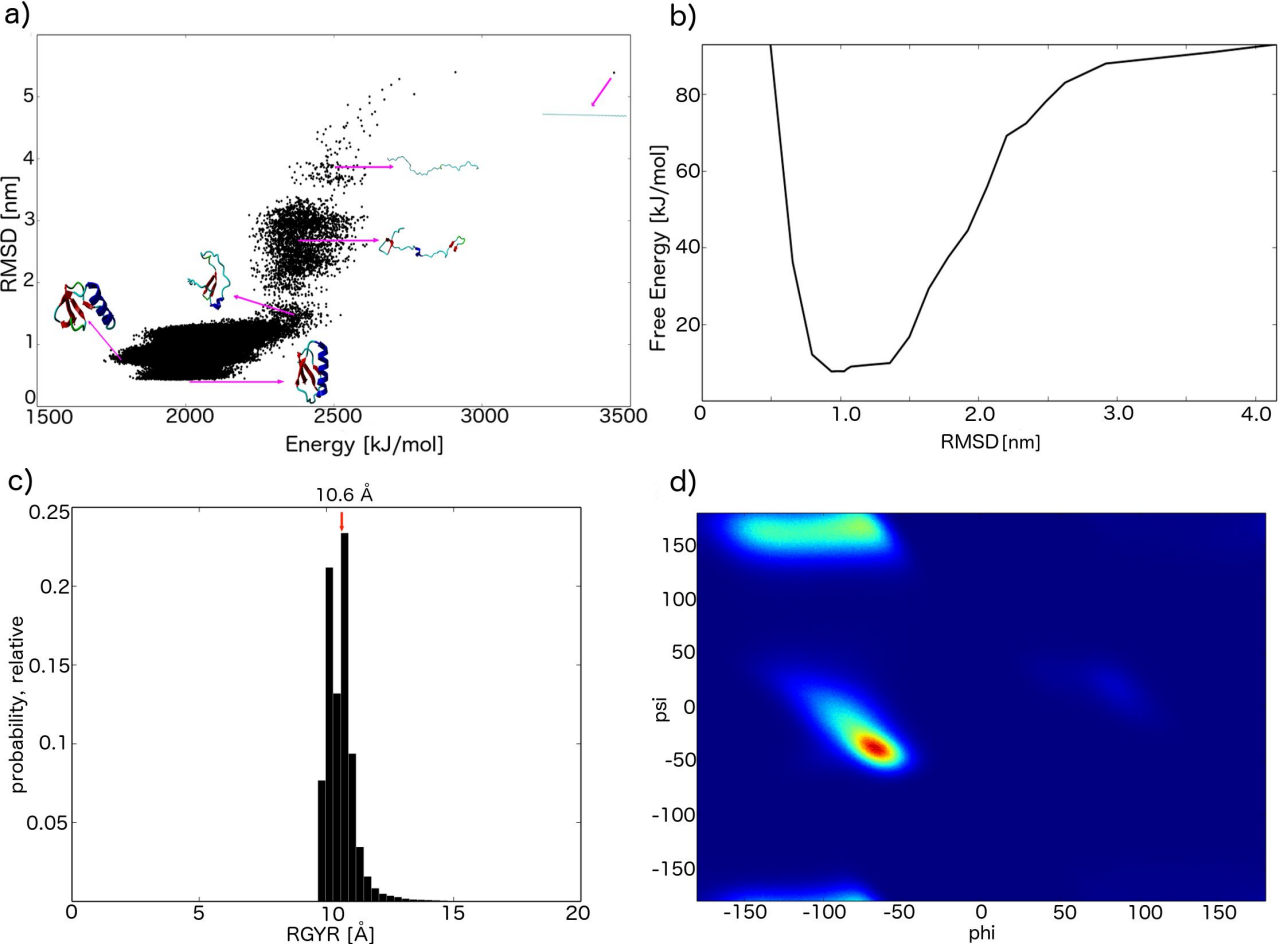
Folding of GB3 using the CamTube force field. (a) CamTube energy generated from an unbiased 1 μs long molecular dynamics simulation of GB3 as a function of the RMSD from the crystal structure, PDB ID: 2OED. Representative structures sampled in different regions of (*energy*, *rmsd*) space are shown as insets. (b) Free energy of Val60 obtained from a metadynamics simulation and the CamTube force field as a function of the RMSD from the crystal structure, PDB ID: 2OED. (c) Distributions of the radius of gyration; the radius of gyration of the native state of GB3 (PDB ID: 2OED) is indicated by the red arrow. (d) Ramachandran plot for the GB3 structures generated by the CamTube force field.

Having identified the conformational space that corresponds to a compact polyamino acid chain (Val60), we turned our attention to the CamTube simulation of a small globular protein. In this respect, we carried out a 1 μs long unbiased molecular dynamics simulation of the third immunoglobulin G-binding domain of protein G (GB3) using *ε*_*W*_ = 10.0 kJ mol^−1^ and *κ*_*c*_ = 2000 kJ mol^−1^ nm^−2^ parameters characteristic of the α/β compact phase. Unlike a homopolymeric peptide chain Val60 that can sample a plethora of protein folds, GB3 is a small globular protein and hence the CamTube model of GB3 is expected to produce a limited repertoire of protein folds. In order to check this hypothesis, the unbiased CamTube simulation of GB3 was initiated from an extended structure and the evolution of sampled structures during the course of the simulation is depicted in Fig 4A. We found that the residual secondary structural content emerged shortly after the start of the simulation. Following the formation of the secondary structure, the protein was able to fold owning to the pairwise hydrophobic energy term in the CamTube force field. After initial folding, the protein was able to rearrange both its secondary structural content and hydrophobic contacts and sample a range of α/β globular folds (Fig 4A). The conformations sampled mainly differ in the fraction of the α-helical and β-sheet content, the number of strands in the β-sheet and the packing of the α-helix against the β-sheet. The topologies of the sampled conformations are similar to that of the native state of GB3 PDB ID: 2OED (TM_score > 0.4) with RMSD values in the range from 4.7 Å to 12 Å. However, given its coarse-grained nature, the present version of the CamTube model was not able to clearly discriminate between the competing minima within this wide basin of native-like topologies, and this point was further corroborated in the free energy surface of the GB3 folding obtained from a metadynamics simulation (Fig 4B).

The results of the quality assessment for the resulting GB3 simulation are shown in Fig 4C and 4D. The structures that we obtained exhibit the distribution of radii of gyration centred on the value characteristic of the native state of GB3 (Fig 4C). These results show that the CamTube samples structures consistent with the compaction of typical protein folds despite the very coarse resolution of modelling the side chains. Moreover, the vast majority of the residues sampled were hydrogen bonded, confirming that the CamTube force field preferentially samples highly ordered structures. Lastly, the Ramachandran plot of the GB3 is strongly biased towards regions of the conformational space typical of folded proteins (Fig 4D). Indeed, the α-helical and β-sheet regions of the Ramachandran space are reproduced rather faithfully because of the directional hydrogen bonding in the CamTube model, and forbidden regions are populated infrequently.

### Concluding remarks

We have described the CamTube force field, which is designed to explore efficiently the conformational space of proteins using molecular dynamics simulations. These simulations can be readily carried out through GROMACS (see Methods). The CamTube model, as other coarse-grained approaches, has the ability to describe the structure, thermodynamics and kinetics of protein folding across a range of time and length scales [30,31,32,33,34,35,36,37,38,39,40]. Similar to other coarse-grained models, however, the interpretation of the time scale in the CamTube model is not straightforward. The main reason is that the underlying energy landscape is smoother as a result of the use of coarse-grained particles. The friction arising from the atomistic degrees of freedom is hence subdued and the effective time sampled using the CamTube model can be several orders of magnitude longer than in the atomistic models.

The development of the CamTube force field was motivated by the observation that the conformational space of proteins can be efficiently navigated by exploiting a tube-like description of polypeptide chains [19,20,21,22]. The CamTube model thus provides a solution to the problem of generating protein-like conformations within coarse-grained simulations without the need of computationally expensive terms in the force field because the underlying geometric nature of the tube already shapes the topological properties of the simulated protein molecules. There will be, however, many applications for which the current implementation of the CamTube model is not well suited, as for instance applications for which long-range electrostatic forces are crucial. For these applications, the residue-residue interactions within the interaction matrix should be turned on. Furthermore, the current implementation is carried out *in vacuo*. However, with relatively small changes in the interaction matrix it will be possible to implement an implicit solvent model.

In conclusion, the use of the CamTube model enables one to explore comprehensively the behaviour of proteins in their native and non-native states with great efficiency, thus providing a wide range of opportunities to study the behaviour of these important molecules. In the long-term, the CamTube is not intended to replace atomistic simulations, but rather to complement them. With the CamTube model the long time-scale and length-scale properties of complex macromolecular systems can be explored, whereas with atomistic models more specific details can be studied. In this view, on the basis of the model presented in this paper, we anticipate that it will be possible to combinef the CamTube with atomistic models in a multi-scale approach.

## Methods

### The CamTube force field: Self-avoiding spheres

**Fig 5:**
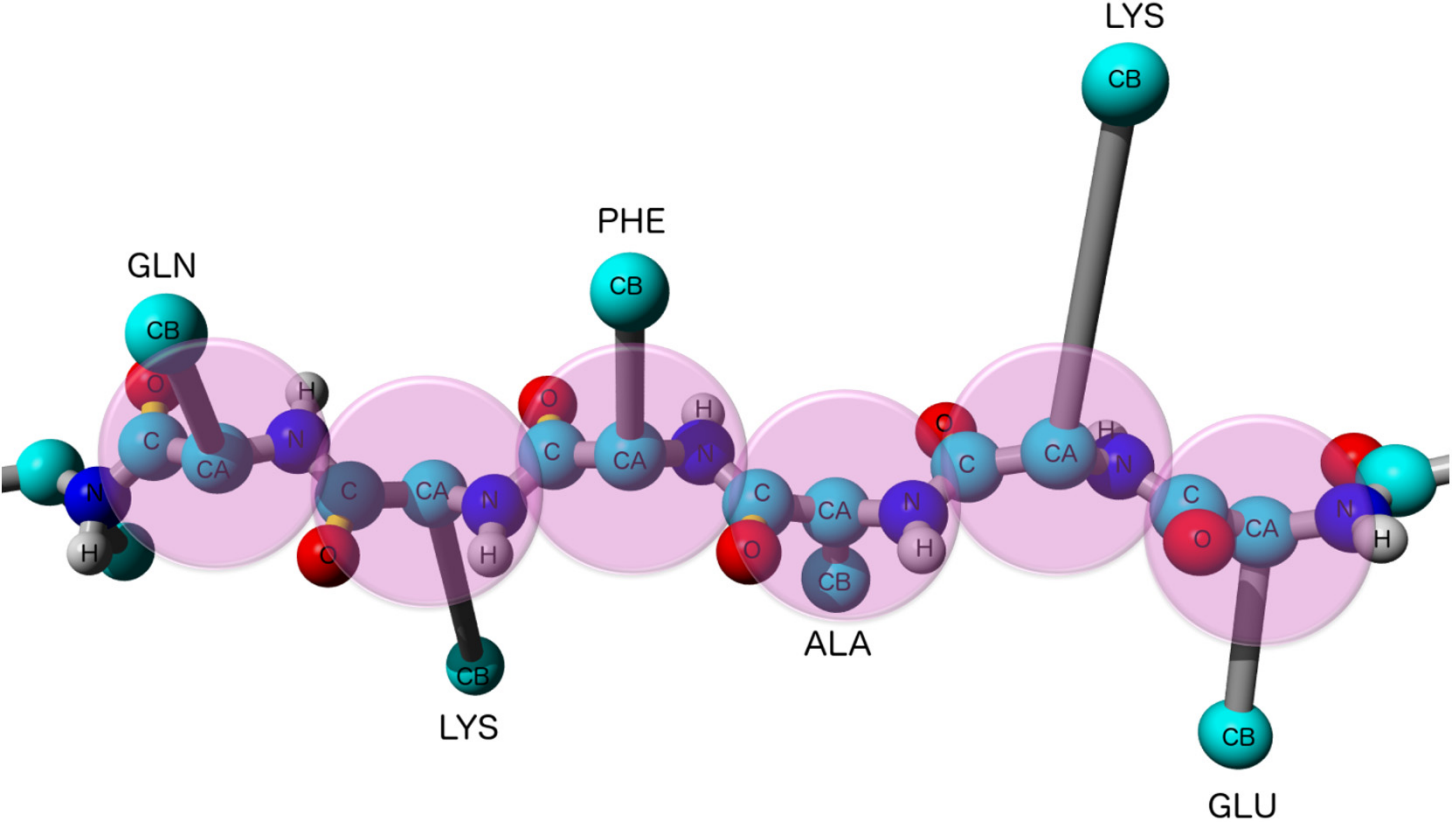
Schematic representation of a segment of a polypeptide chain in the CamTube model. The tube-like implementation is carried out by self-avoiding spheres, which for clarity of illustration are shown here only for Cα atoms. Bond lengths (apart from the Cα-Cβ bond) and angles are taken from the Amber force field. The length of the CA-Cβ bond of Val, Pro, Thr, Ser and Cys is scaled 1.5 times; Asp, Ile, Leu and Asn 2 times; Phe 2.25 times; Glu, Gln, Met and His 2.5 times; Tyr and Trp 3 times; Lys and Arg 4 times the length of the Cα-Cβ bond in the Amber force field.

The CamTube force field is defined by five backbone (N, H, Cα, C′ and O) and one side chain (Cβ) atom types in a polypeptide chain. The bonded interactions in the model, including covalent bonds and angles are taken from the Amber force field [41]. The lengths of the Cα-Cβ bonds are amino acid-dependent in the CamTube model and overwrite the standard parameters from the Amber force field (Fig 5). The bond lengths are constrained using LINCS [42], and a 2 fs time step is used to integrate the equations of motion.

**Fig 6:**
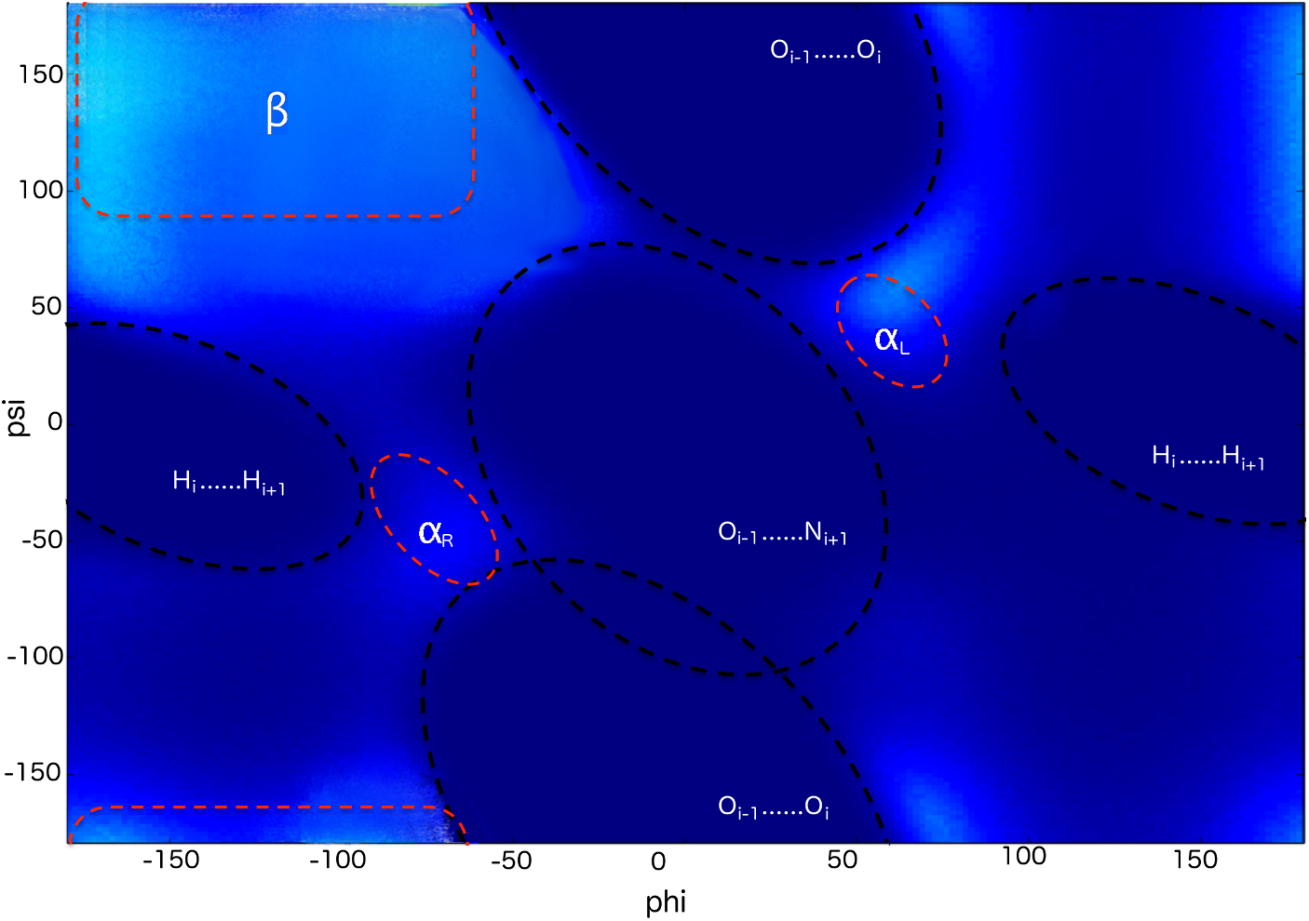
Steric map in the CamTube model. The map shows main steric restrictions (dashed black line) imposed by H_i_-H_i+1_, O_i–1_-O_i_ and O_i–1_-N_i+1_ distances. Allowed regions are represented by light blue colour and they contain the range of dihedral angles present in right-handed α-helices, left-handed α-helices and β-sheets.

The tube-like characteristics of the polypeptide chain are retained via the self-avoiding spheres centred on the atom positions. The radii of the spheres are atom pair-dependent and their role is to exclude the regions of the Ramachandran map that encode steric clashes. The interactions between such hard spheres are modelled as a half harmonic potential:

$$E_{ij}^{tube}=\left\{ \begin{array}{l} \kappa_{tube}{\left(d-r_{ij}\right)}^{2}\vartheta (d-r_{ij})\;|i-j|>1\\ 0\;|i-j|=1 \end{array}\right.$$ (1)

where *ϑ(x)* is the Heaviside step function, *d* is an atom pair dependent distance threshold and *r*_*ij*_ is the distance between the atoms of residues *i* and *j*. The parameter *κ*_*tube*_ is chosen to be as large as possible without introducing integration errors or numerical instabilities, so as to guarantee the impenetrability of the sphere. In the force field setup described here, the 2 fs integration time step allows us to take *κ*_*tube*_ = 100,000 kJ mol^−1^ nm^−2^, which is comparable to the largest force constants in the Amber bonded potentials. The atom pair-dependent distance thresholds, *d*, are obtained from the statistical distribution from the PDB [43,44]. The values of 5% minimum (5^th^ percentile band) are used here and listed in Table 1. Nearest neighbours are not interacting through this potential for all atom pairs apart from O-O and H-H pairs. The O-O and H-H nearest neighbour pairs are crucial in excluding forbidden regions in the Ramachandran map [43,44] and they are retained in the CamTube model. The steric map of the CamTube model showing steric restrictions (dark blue) and sterically allowed regions (light blue) is given in Fig 6, and illustrates how sterically allowed regions contain the areas of the Ramachandran map characteristic of right-handed α-helices, left-handed α-helices and β-sheets.

**Table 1:** Atom pair self-avoiding sphere distances, *d*, from Eq 1.

Atom types	Threshold [nm]	Atom types	Threshold [nm]
H-H	0.23	Cβ-Cβ	0.4
O-O	0.3	Cβ-C	0.32
Cα-Cα	0.4	Cβ-H	0.26
Cα-Cβ	0.4	Cβ-N	0.32
Cα-C	0.35	Cβ-O	0.3
Cα-H	0.30	C'-C'	0.35
Cα-N	0.35	C'-O	0.3
Cα-O	0.3	N-N	0.36

### The CamTube force field: Hydrogen bonding

Hydrogen bonds in the CamTube force field are modelled using a potential between pairs of O and H atoms of the Lennard-Jones form:

$$E_{ij}^{H-bond}=\left\{ \begin{array}{l} \epsilon_{H}[\frac{n}{m-n}{\left(\frac{r_{0}}{r_{ij}}\right)}^{m}-\frac{m}{m-n}{\left(\frac{r_{0}}{r_{ij}}\right)}^{n}]\;|i-j|\geq 4\\ 0\;|i-j|<4 \end{array}\right.$$ (2)

where *r*_*ij*_ is the distance between the O atom of residue *i* and H atom of residue *j*, and *r*_*0*_ is taken to be an ideal hydrogen bond length of 0.2 nm.

The functional form in Eq 2 defines a smooth potential with an attractive basin at *r = r*_*0*_ and a well depth of *ε*_*H*_ for any positive exponents *m ≠ n*. The choice of exponents is constrained by two requirements. The first is that the potential should preferentially promote each H or O atom to participate to a single hydrogen bond. This means that the potential must decay rapidly to 0 for *r > r*_*0*_ in order to prevent extensive crosstalk between nearby hydrogen bonds. The second requirement is that, at the same time, the potential must be sufficiently attractive at large distances in order for potential hydrogen bonding partners to come together in the first place. This requirement also ensures that the potential is soft enough to allow hydrogen bonds to form readily, allowing the tube model to rapidly cycle through different tertiary motifs.

We tested the 6–12, 10–12 and 12–24 variants of the *n-m* potential and found that the 10–12 version provides the best compromise between confinement of the attractive basin and kinetic accessibility. The well depth *ε*_*H*_ sets the fundamental energy scale of the system, and all other energy parameters in the model are tuned relative to this value. For a given temperature and sampling method, the choice of *ε*_*H*_ can dramatically affect the observed kinetics. For large values of *ε*_*H*_ the polypeptide chain is unable to escape from local minima, which for small values of *ε*_*H*_ the secondary structure elements are not stable enough to enable the protein to fold. We found that in the case of Langevin dynamics *in vacuo* at a temperature of 298 K and a friction coefficient of 1 ps^−1^, an efficient compromise between these two extreme behaviours was reached using a well depth of 21 kJ mol^−1^.

### The CamTube force field: Directionality of hydrogen bonds

**Fig 7:**
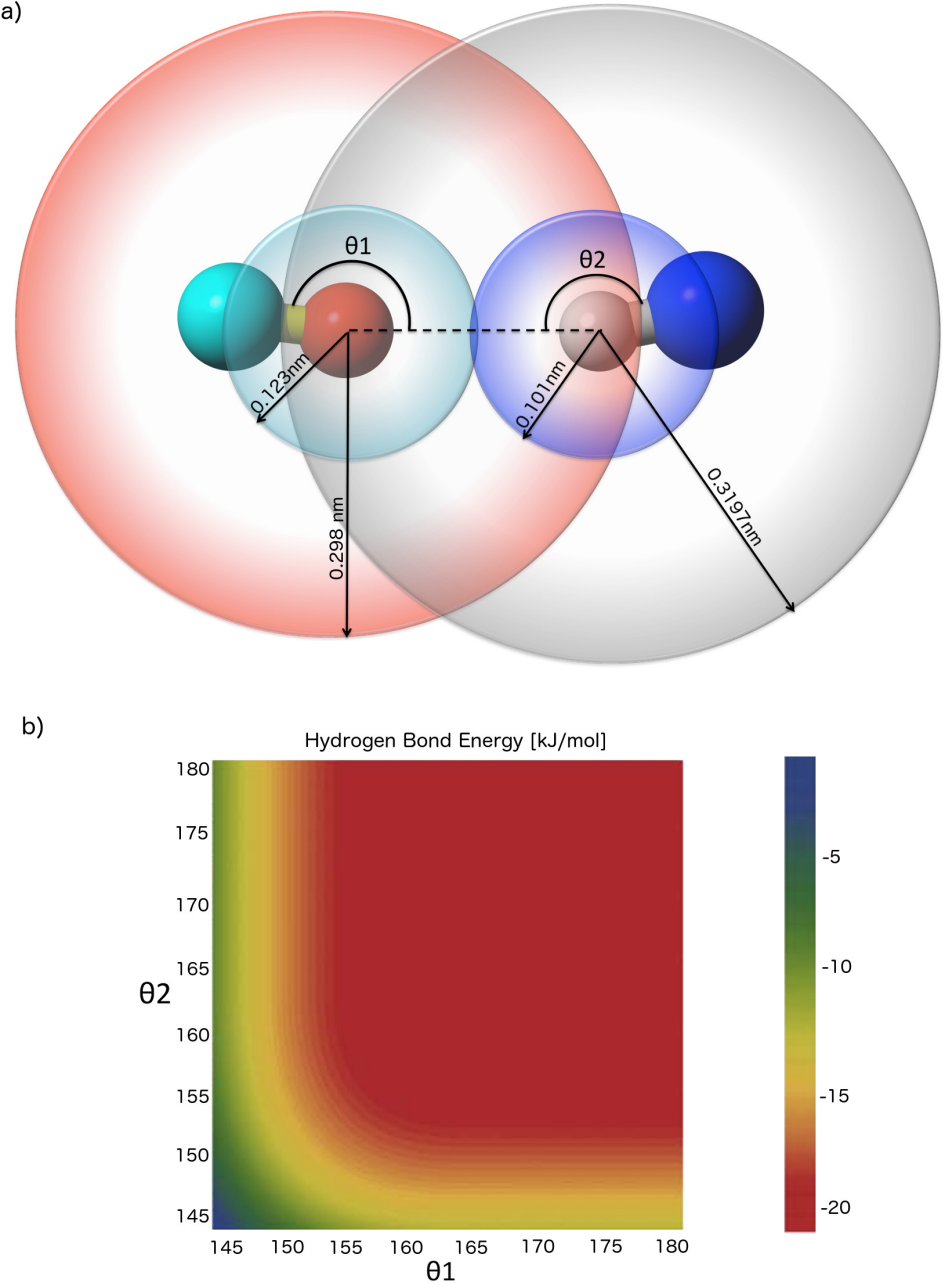
Illustration of the directionality of the hydrogen bonds in the CamTube model. (a) The use of spherical avoidance volumes prohibits bond angles far from 180°. The C', O, H, and N atoms are shown in teal, red, grey, and blue, respectively. (b) Angular dependence of the overall hydrogen bonding potential after the inclusion of half harmonic repulsions between C'-H and O-N pairs. The potential is plotted at the optimal O-H distance of 0.2 nm using *ε*_*H*_ = 21 kJ mol^−1^.

Unlike atomistic force fields, which can induce the formation of directional hydrogen bonds via a combination of steric and electrostatic interactions, the CamTube coarse-grained setup discussed here requires a more direct introduction of this directionality. We addressed this issue in an analogous manner to the tube geometry by placing spherical avoidance volumes between all C'-H and O-N atom pairs (Fig 7A, red and grey spheres). The radii of these spheres are chosen to correspond to 99% of the C'-H or O-N distance in an ideal hydrogen bond with 180° bond angles. As a result, when an O-H atom pair forms a hydrogen bond at the ideal distance of 0.2 nm, a combination of these self-avoidance spheres and the fixed C'-O and N-H bond lengths constrains the allowed positions of the C' and N atoms and prevents large deviations from the ideal hydrogen bond angles. For example, in the hydrogen bond shown in Fig 7A, the N atom is constrained to lie on the subset of the blue spherical surface that does not fall inside the red sphere. Likewise, the C' atom is confined to the regions of the teal spherical surface that are outside the grey sphere.

As in Eq 1, we implemented the avoidance spheres of hydrogen bonds as half harmonic potentials, with large force constants *κ*_*tube*_ = 100,000 kJ mol^−1^ nm^−2^ to ensure impenetrability:

$$E_{ij}^{CH}=\left\{ \begin{array}{l} \kappa_{tube}{\left(0.3197-r_{ij}^{CH}\right)}^{2}\vartheta (0.3197-r_{ij}^{CH})\;|i-j|\geq 4\\ 0\;|i-j|<4 \end{array}\right.$$ (3)

$$E_{ij}^{ON}=\left\{ \begin{array}{l} \kappa_{tube}{\left(0.2980-r_{ij}^{ON}\right)}^{2}\vartheta (0.2980-r_{ij}^{ON})\;|i-j|\geq 4\\ 0\;|i-j|<4 \end{array}\right.$$ (4)

Since the C'-O and N-H bond lengths are fixed by LINCS, these potentials can be rewritten as functions of the C'-O-H and O-H-N angles using the law of cosines. The resulting angular dependence of the overall hydrogen bond energy is shown in Fig 7B, verifying that highly bent hydrogen bonding geometries are energetically unfavourable.

### The CamTube force field: Hydrophobic effect

In a previous implementation of the tube model [22], the hydrophobic effect was simulated using the HP (hydrophobic-polar) propensities of the amino acids with a square well attraction between hydrophobic residues. This model was later extended by incorporating a residue-specific contact potential [24], which was adapted from lattice models of protein folding and parameterised based on the contact frequencies between amino acid pairs in the PDB [45]. This residue-specific matrix has as a reference state the interaction between the solvent and all other amino acids that is set to 0 and hence is suitable for *in vacuo* simulations.

Given the success of this type of pair potential in allowing the sequence design of tertiary structures, we adopted a similar model to efficiently sample the conformational space in a sequence-specific manner. Thus, the CamTube force field features an analogous residue-specific interaction potential that is used here only between the Cβ atoms of the hydrophobic residue pairs:

$$E_{ij}^{hydrophobic}=\left\{ \begin{array}{l} \epsilon_{w}B_{ij}[1-\frac{1}{1+exp(\frac{0.8-r_{ij}}{0.04})}]\;|i-j|>1\\ 0\;|i-j|=1 \end{array}\right.$$ (5)

where *r*_*ij*_ is the distance between the Cβ atoms of the residues *i* and *j* and *B*_*ij*_ is the element corresponding to the particular amino acid pairing. The values of *B*_*ij*_ for a pair of hydrophobic residues are taken from the interaction matrix [45], and set to 0 if either *i* or *j* is a polar or charged amino acid. In this way, the conformation of a protein is mainly defined by the interplay between hydrogen bond and hydrophobic interactions. The parameter *ε*_*W*_ sets the energy scale for hydrophobic contacts. The consequences of different values of *ε*_*W*_ on the structures sampled by the CamTube force field are discussed in the Results section.

### The CamTube force field: Curvature penalty

**Fig 8:**
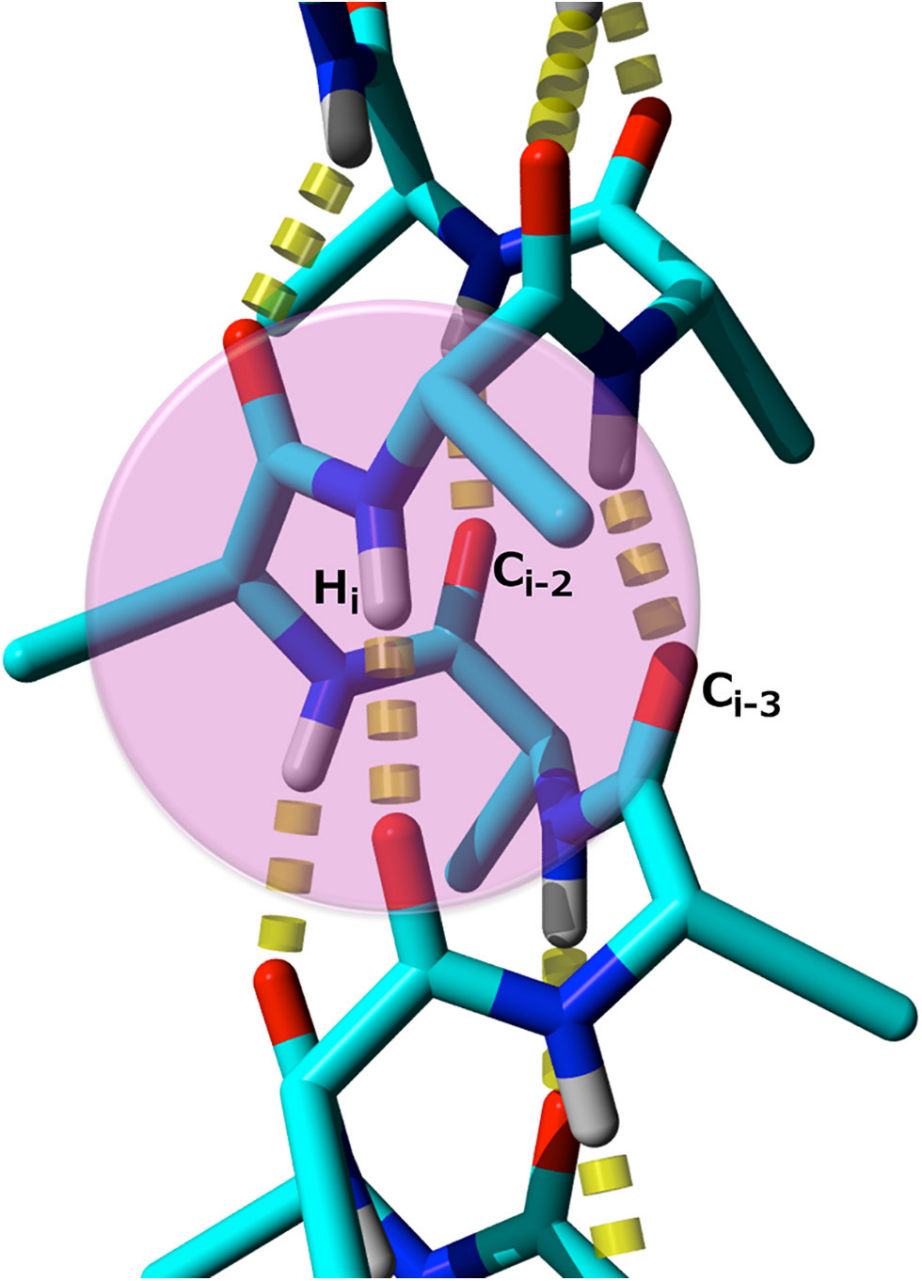
Illustration of the repulsion between C' and H atoms introduced by the curvature term in the CamTube model. The C' and H atoms belong to the α-helix and are 2 and 3 residues apart in the sequence.

The ability of the tube model to visit protein-like conformations is dependent on the presence of geometric constraints that eliminate non-physical topologies of the tube from the accessible conformational space. In the previous Monte Carlo implementation of the tube model [22], this result was accomplished by means of a weak penalty on the local radius of curvature of the tube. We have translated this energy penalty into a form suitable for molecular dynamics simulations by introducing a weak repulsive force between C' and H atoms that are separated by two or three residues along the protein chain (Fig 8). For ease of implementation in Gromacs, we used the same functional form for these repulsions as for the directional hydrogen bonds (Eqs 3 and 4), but with a sufficiently weak force constant to transform the potential from a hard constraint into a soft penalty:

$$E_{ij}^{curv}=\left\{ \begin{array}{l} \kappa_{c}{\left(0.3197-r_{ij}^{CH}\right)}^{2}\vartheta (0.3197-r_{ij}^{CH})\;1<|i-j|<4\\ 0\;\text{otherwise} \end{array}\right.$$ (6)

The presence of this curvature term has a main effect on interactions between C' and H atoms that are separated by two or three residues in α-helix. When the interactions between these atoms occur at distances below the threshold of 0.3197 nm, the curvature term becomes active. This means that the formation of α-helical segments can be marginally penalized by tuning the value of *κ*_*c*_, which is essential to prevent all sequences from immediately adopting extended α-helical structures with exclusively local hydrogen bonding.

### The CamTube force field: Dihedral potentials

The purpose of the dihedral parameters in the CamTube force field is to cluster the conformations of amino acids near their characteristic values in the Ramachandran plot [46]. Almost all dihedral and improper parameters were taken from the Amber force field [41]. Only residue-specific Cβ-Cα-N-C' and Cβ-Cα-C'-N dihedral parameters are refitted for the purposes of the CamTube model and their values overwrite the corresponding values in the Amber force field. The parameters for Cβ-Cα-N-C' and Cβ-Cα-C'-N dihedrals are refitted here using the following values for hydrogen bond potential, hydrophobic potential and curvature penalty: e_H_ = 21 kJ mol^−1^, e_w_ = 10 kJ mol^−1^ and k_c_ = 2000 kJ mol^−1^ nm^−2^ (see Phase diagram section). The fitting procedure was carried out using a full combinatorial parameter scan that varied the Vn parameters on the grid from 0.0 to 10.0, with the step size of 0.25.

**Fig 9:**
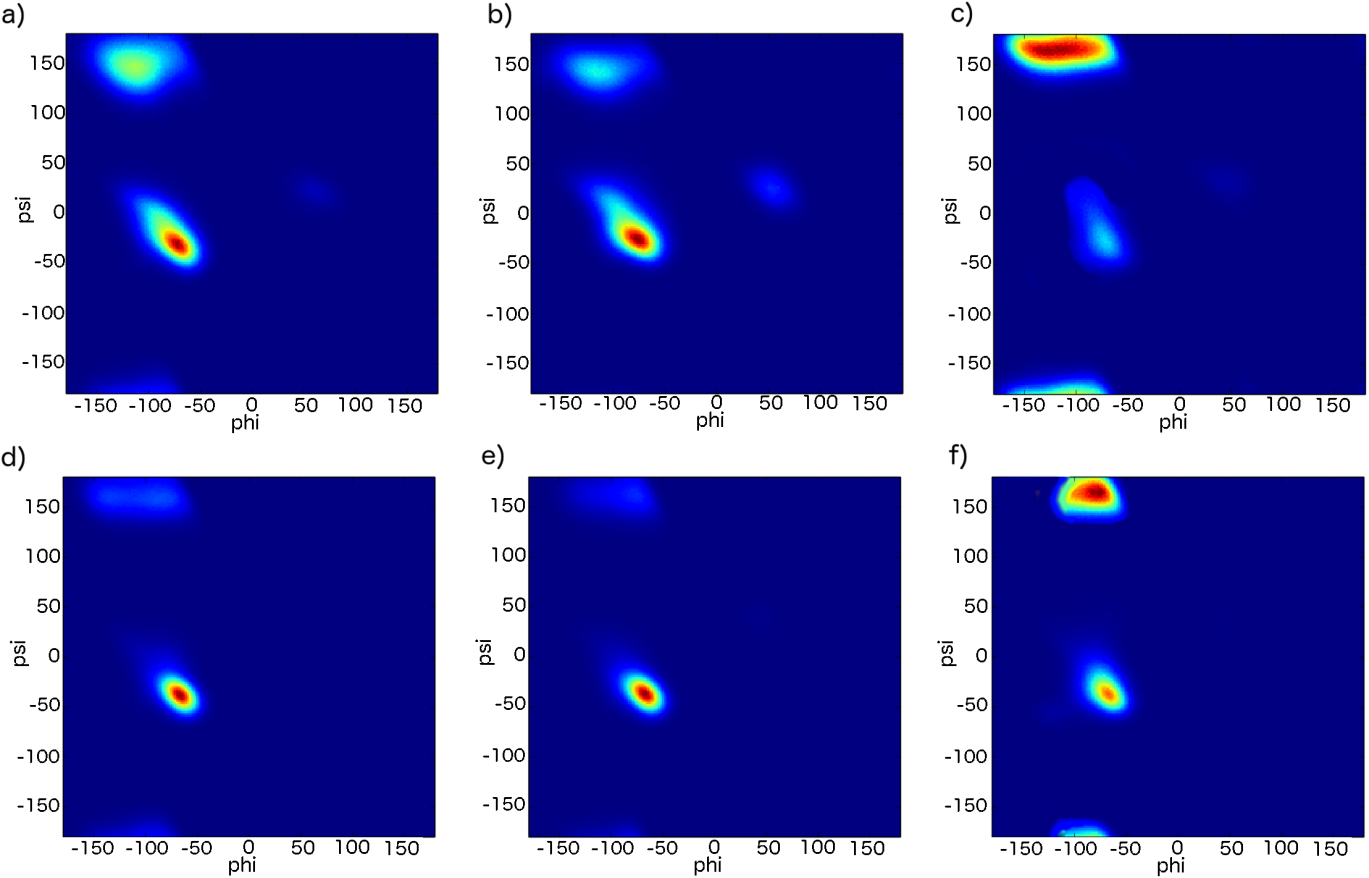
Ramachandran maps of non-Gly residues after the introduction of the dihedral potentials in the CamTube force field. Residues are grouped according to their propensity for particular regions in the Ramachandran map: a) Arg, Cys, Met, Leu, Ser, Trp; b) Asn, Asp, His; c) Ile, Phe, Thr, Tyr, Val; d) Ala, Gln; e) Glu, Lys; f) Pro.

Obtained residue-specific values are listed in Table 2 and their corresponding Ramachandran maps are depicted in Fig 9. The dihedral parameters of amino acids are grouped depending on their characteristic propensities to α-helical and β-sheet regions of the Ramachandran map obtained from the PDB [46]. Ile, Thr, Tyr, Phe and Val are similar in their unique property of preferring the β-sheet to α-helix conformation and are assigned identical distributions in the Ramachandran map. His, Asn and Asp have quite similar distributions with non-negligible populations of left-handed α-helix. Their Ramachandran maps reflect the role of especially Asp and Asn in terminating α-helices and β-sheets. Ala, Gln, Lys and Glu are similar in preferring α-helix to β-sheet, whereas Pro has a bimodal distribution of α-helix and polyproline II conformation. Finally, Arg, Cys, Leu, Met, Ser and Trp are assigned identical distribution due to their almost equal propensity for both α-helices and β-sheets.

**Table 2:** Parameters for Cβ-Cα-N-C' and Cβ-Cα-C'-N dihedral angles used in the CamTube force field that encode the propensity of the amino acids for different regions in the Ramachandran map.

Amino acid	Angle definition	Function type	Vn	γ	n
Arg, Asn, Asp, Cys, His, Ile, Leu, Met, Phe, Ser, Thr, Trp, Tyr, Val	Cβ Cα N C	9	10.0	0	1
	Cβ Cα N C	9	5.0	0	2
	Cβ Cα N C	9	0.0	0	3
Ala, Gln, Glu, Lys, Pro	Cβ Cα N C	9	20.0	0	1
	Cβ Cα N C	9	10.0	0	2
	Cβ Cα N C	9	2.0	0	3
Arg, Cys, Glu, Leu, Lys, Met, Ser, Trp	Cβ Cα C N	9	1.0	0	1
	Cβ Cα C N	9	2.5	0	2
	Cβ Cα C N	9	1.0	0	3
Asn, Asp, His, Pro	Cβ Cα C N	9	10.0	0	1
	Cβ Cα C N	9	5.0	0	2
	Cβ Cα C N	9	2.5	0	3
Ala, Gln, Ile, Phe, Thr, Tyr, Val	Cβ Cα C N	9	0.0	0	1
	Cβ Cα C N	9	2.5	0	2
	Cβ Cα C N	9	2.5	0	3

### Implementation of the CamTube force field

The CamTube model described above was implemented in Gromacs via a suite of Python scripts. This toolkit includes a script that mimics the functionality of the native *pdb2gmx* utility in Gromacs by parsing an input PDB file, removing side chains and adding Cβ and H atoms as necessary, constructing a topology file using the relevant bonded parameters from the Amber force field, adding pair lists and exclusions for consistency with Eqs 1–6, and preparing an index file with designated index groups for all six-atom types.

The CamTube potential energy surface is implemented as a series of tabulated potentials that are activated by specifying *coulombtype* and *vdwtype* as ‘User’ in the *mdp* file. Simulations with CamTube are set up with the *grompp* utility as with any other Gromacs force field, and are carried out by supplying the tabulated potentials as inputs to the *mdrun* program. The set of *mdp* parameters that we have used in the development and testing of the force field is shown in Table 3. Using these parameters, *in vacuo* simulations of a 60-residue peptide can be run at a rate of over 1 μs/day on a laptop computer with a 2.3 GHz quad-core i7 processor.

**Table 3:** Input parameters used in simulations with the CamTube force field.

integrator	sd
dt	0.002
comm-mode	Angular
nstcomm	1
energygrps	Cα Cβ C O N H
nstlist	-1
ns-type	simple
pbc	no
rlist	1.5
coulombtype	User
rcoulomb	0.8
vdwtype	User
rvdw	1.2
table-extension	0.5
energygrp-table	Cα Cα Cβ Cβ Cα Cβ O O H H C H O H O N C Cβ H Cβ N Cβ O Cβ C Cα H Cα N Cα O Cα O C C C N N
tau-t	1.0
ref-t	298
constraints	all-bonds
constraint-algorithm	LINCS
lincs-order	6
lincs-iter	2

### Bias-exchange metadynamics simulations of Val60 and GB3

Bias-exchange metadynamics simulations of Val60 and GB3 were performed at 300K using four replicas, one for each of the collective variables (CVs):

1) CV1 (dihedral correlation) acts on ϕ and ψ dihedral angles of all residues in the protein, parameters: Gaussian width σ = 0.1; 2) CV2 (radius of gyration) acts on the radius of gyration defined by the Cα, atoms, parameters: Gaussian width σ = 0.05; 3) CV3 (AlphaRMSD) counts the number of 6-residue fragments that have α-helical secondary structural content, parameters: Gaussian width σ = 0.5; 4) CV4 (AntiBetaRMSD) counts the number of 6-residue fragments that have antiparallel β-sheet secondary structural content, parameters: Gaussian width σ = 0.5. The functional forms of the CVs are defined in the PLUMED package [47]. Starting from an extended conformation of both Val60 and GB3 in vacuum, we run 5μs for each replica using the CamTube force field.

### Free energy reconstruction in the CV space

The bias potentials became stable after a simulation time t_eq_ = 0.5 μs for Val60 and t_eq_ = 0.32 for GB3. The simulations were further run up to 5 μs in order to reconstruct the free-energy landscape of the proteins. The configurations were then grouped together in microstates by dividing the 4-dimensional CV-space into a grid of small hypercubes [27,48]. Each frame of the trajectory was assigned to the hypercube to which it belongs and the set of frames contained in a hypercube defined a microstate. The relative free energies of each microstate were corrected using the effect of the bias. They were estimated by a WHAM approach as described previously [48] using METAGUI [27] and Visual Molecular Dynamics (VMD) [49] interface. In total, 1451 and 1707 microstates and corresponding free energies were identified for Val60 and GB3, respectively.

### CamTube availability

The CamTube force field is publicly available through GROMACS, with also scripts available in PLUMED. [47].
